# Effect of mol­ecular perturbation on co­crys­tal formation: theo­phyl­line and its 8-halo analogues with flavonoids

**DOI:** 10.1107/S2053229626006637

**Published:** 2026-07-07

**Authors:** Weijian Ye, Herman H-Y. Sung, Ian D. Williams

**Affiliations:** a Department of Chemistry, The Hong Kong University of Science and Technology, Clear Water Bay, Hong Kong SAR, People’s Republic of China; J-PARC Center, Japan Atomic Energy Agency, Japan

**Keywords:** crystal structure, co­crys­tal, alkaloid, flavonoid, theo­phyl­line, hydrogen bond, myricetin, kaempferol

## Abstract

The tendency for co­crys­tal formation between alkaloids and flavonoids is found to be quite common (around 30%) and based on the optimization of hy­dro­gen-bond formation between the acceptor-rich alkaloids and donor-rich flavonoids. Within each mol­ecular family there is substantial variation in co­crys­tal tendency, leading to considerable scope for selective co­crys­tal precipitation to assist in the separation of com­plex mixtures of these natural product families. Theophylline (C_7_H_8_N_4_O_2_) follows caffeine and theacrine in forming a substantial number of flavonoid co­crys­tals – six in total, of which three were reported previously. By contrast, the 8-halo-substituted analogues 8-*X*-Tph [*X* = Cl (C_7_H_7_ClN_4_O_2_), Br (C_7_H_7_BrN_4_O_2_)] form co­crys­tals with just myricetin (C_15_H_10_O_8_) and kaempferol (C_15_H_10_O_6_). Weakening of the acceptor capability of the alkaloid N9 atom upon proximal halo substitution may play a role in the reduced tendency of 8-*X*-Tph towards co­crys­tal formation, since improved packing efficiency is not a key factor in the formation of these co­crys­tals. Most of the co­crys­tal phases may be conveniently prepared with good yield and purity through either liquid-assisted grinding (LAG) or microwave-assisted co­crys­tallization from 1-butanol.

## Abstract 2

The tendency for alkaloid–flavonoid co­crys­tal formation is found to be markedly different for theo­phyl­line (Tph) com­pared to its 8-halo analogues. Through screening with 13 common flavonoids, Tph is found to form co­crys­tals with six flavonoids, whereas 8-Cl-Tph and 8-Br-Tph each form co­crys­tals with just two, myricetin and kaempferol. The weakening of the alkaloid N9 atom as a hy­dro­gen-bond acceptor towards the hy­dro­gen-bond-donor-rich flavonoids may play a role in this differing tendency.

## Introduction

The formation of co­crys­tals of natural products has been of considerable recent inter­est. For those mol­ecules with bioactivity that can be considered as pharmaceuticals, there is the associated inter­est of potential improvement in oral delivery, enhanced dissolution, bioavailability, solid-state stability, mechanical properties, tabletability, hygroscopic properties and avoidance of hydrate formation (Trask *et al.*, 2005 ▸; Good & Rodríguez-Hornedo, 2009 ▸; Chadha *et al.*, 2017 ▸; Zhu *et al.*, 2017 ▸). Cocrystals may also have important intellectual property implications (Sharma *et al.*, 2025 ▸). The report that co­crys­tal formation between caffeine and various flavonoids could lead to their enhanced separation *via* green technology based on differential solubility of the co­crys­tals was intriguing (Xia *et al.*, 2021 ▸). If such natural product mixtures could be separated based on dif­ferences in co­crys­tal solubility, how much more effective if the mixture was subject to a differential tendency to form such co­crys­tals in the first place? We thus investigated the general tendency of alkaloids to form crystals with flavonoids in a survey of co-former pairs. This was done relatively efficiently by screening for co­crys­tal formation *via* a liquid-assisted grinding (LAG) ap­proach (Trask & Jones, 2005 ▸). This was helpful in screening for co­crys­tal formation between the highly potent anti­malarial agent 11-aza­ar­tem­is­in­in with a variety of coformers, including many carb­oxy­lic acids (Nisar *et al.*, 2018 ▸; Roy *et al.*, 2021 ▸; Li *et al.*, 2022 ▸). Successful co­crys­tal formation is quickly checked by running powder X-ray diffractograms of the resulting powders after co-grinding for several hours. This practically ensures that for successful combinations, when a change in the powder X-ray diffraction (PXRD) pattern is observed, the resulting co­crys­tals represent thermodynamically preferred solid forms.

In order to find the structure and stoichiometry of such co­crys­tal products, a rational and consistent crystallization ap­proach was also sought that could be rapidly tested and applied to successful coformer pair combinations. After various efforts, a standard methodology of heating/cooling aqueous methano­lic solutions over a period of 6–12 h was adopted. The result for around 250 total combinations of common alkaloids and common flavonoids is that 75 co­crys­tal phases were readily isolable and have been characterized by single-crystal X-ray structure determination (Ye, 2024 ▸).

**Figure 1:**
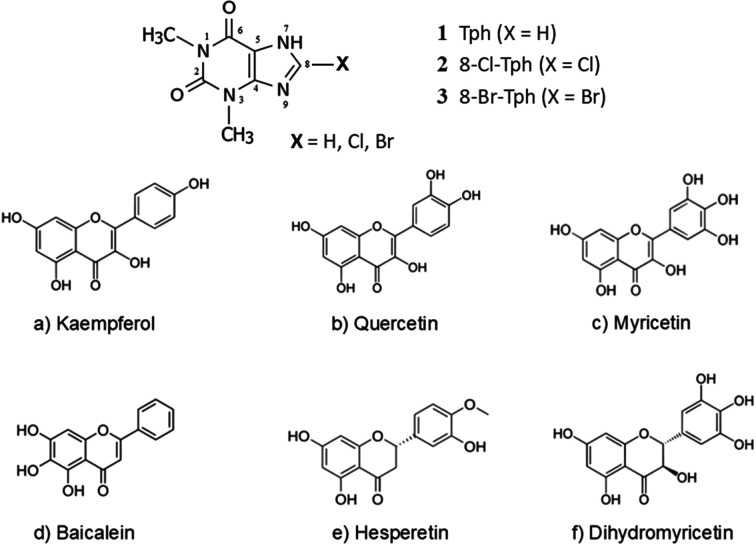
Structural scheme for theo­phyl­lines (**1**–**3**) and flavonoids (**a**–**f**) used in the co­crys­tal phases.

In this article, we report the com­parative results of co­crys­tal formation with flavonoids for the alkaloid theo­phyl­line com­pared to its 8-halo analogues (Fig. 1 ▸). Recently, we reported the crystal structures of 8-Cl-Tph and 8-Br-Tph, and found four unreported forms that were structurally distinct from the polymorphs of the parent theo­phyl­line (Ye *et al.*, 2025 ▸). The marked switch of structure types appeared strongly linked to the electronic modification of the Tph mol­ecule upon introduction of the 8-halo substituent. This renders the resulting protonated cations [Tph-H]^+^ more acidic, but also lowers the basicity of the ring N9 atom that is adjacent to the halo substitution site. This apparently leads to a switch of polymorph hy­dro­gen-bond preference from N—H⋯N found in most Tph polymorphs to N—H⋯O found in 8-*X*-Tph. We were curious to explore whether this electronic perturbation, which was supported by our DFT calculations, might also effect co­crys­tal formation for these related alkaloids.

The co­crys­tals reported here are: theo­phyl­line–kaempferol (1/1) (**1a**), theo­phyl­line–myricetin–water (2/2/1) (**1c**), theo­phyl­line–*rac*-hesperetin (2/1) (**1e**), 8-chloro­theo­phyl­line–kaempferol (1/1) (**2a**), 8-chloro­theo­phyl­line–myricetin (1/1) (**2c**), 8-bromo­theo­phyl­line–kaempferol (**3a**), 8-bromo­theo­phyl­line–myricetin (1/1) (**3c**), 8-bromo­theo­phyl­line–kaempferol–methanol (1/1/1) (**3a′**). We also report the previously unpublished structure of kaempferol monohydrate (**a·H_2_O**).

## Experimental

### Isolation and crystallization

#### General

The theo­phyl­line alkaloids (**1**–**3**), flavonoids (**a**–**f**) (Fig. 1 ▸) and solvents used were of reagent grade supplied by Meryer Chemicals or TCI Chemicals (Shanghai) with the following details: (**1**) theo­phyl­line, 99%, CAS 58-55-9, C_7_H_8_N_4_O_2_, *M*_r_ 180.17; (**2**) 8-chloro­theo­phyl­line, >98%, CAS 85-18-7, C_7_H_7_ClN_4_O_2_, *M*_r_ 214.61; (**3**) 8-bromo­theo­phyl­line, 97%, CAS 10381-75-6, C_7_H_7_BrN_4_O_2_, *M*_r_ 259.06. Flavonoids resulting in the successful isolation of co­crys­tals are shown in Fig. 1 ▸ and are: (**a**) kaempferol hydrate, 98%, CAS 520-18-3, C_15_H_10_O_6_·*x*H_2_O, *M*_r_ 286.24 (anhydrous); (**b**) quercetin dihydrate, 97%, CAS 6151-25-3, C_15_H_10_O_7_·2H_2_O, *M*_r_ 338.27; (**c**) myricetin, 97%, CAS 529-44-2, C_15_H_10_O_8_, *M*_r_ 318.24; (**d**) baicalein, 98%, CAS 491-67-8, C_15_H_10_O_5_, *M*_r_ 270.24; (**e**) *rac*-hesperetin, >97%, CAS 520-33-2, C_16_H_14_O_6_, *M*_r_ 302.28; (**f**) *rac*-di­hydro­myricetin, 97%, CAS 27200-12-0, C_15_H_12_O_8_, *M*_r_ 320.25. The use of the flavonoids fisetin, luteolin, genistein, naringenin, biochanin a, chrysin or rutin was unsuccessful for co­crys­tal formation with these theo­phyl­line alkaloids.

#### Cocrystal screening and growth

Screening for co­crys­tallization between alkaloids 8-*X*-Tph (*X* = H, Br, Cl) and flavonoids was carried out by the LAG of 1:1 mixtures (Trask & Jones, 2005 ▸). Grinding used a Tencan XQM-0.4A mini-planetary ball mill with zirconia vessels and media (Nisar *et al.*, 2018 ▸). A minimal amount of methanol (η factor = 0.2 ml g^−1^) was used to accelerate the solid-state transformations, which typically took between 30 min and 2 h.

Single crystals of alkaloid–flavonoid co­crys­tal phases were also grown by a standard ap­proach of solvothermal co­crys­tallization (Karimi-Jafari *et al.*, 2018 ▸) using 0.25 mmol each of the alkaloid (45–65 mg) with the flavonoid (70–85 mg) in aqueous methanol com­prising 2 ml MeOH and 0.1 ml H_2_O. Solutions were heated in 23 ml Teflon-lined Parr-type pressure vessels at 120 °C and under autogenous pressure for 12–24 h, followed by slow cooling back to ambient conditions. Solids were filtered off, washed with cold water and ethanol, dried and inspected under an optical microscope. The co­crys­tal phases prepared in this manner had typical isolated yields of 40–60 mg (65–85% yield based on the limiting reagent).

All co­crys­tals displayed a 1:1 stoichiometry except for theo­phyl­line–baicalein (2:1) and theo­phyl­line–hesperetin (2:1). A previously reported theo­phyl­line–di­hydro­myricetin 1:1 ACN solvate phase from aceto­nitrile (Sun *et al.*, 2023 ▸) was prepared successfully from a modification of the co­crys­tallization solvent to 1:1 MeCN–H_2_O. No change of PXRD pattern emerged in this system upon LAG or solvothermally using MeOH. Finally, co­crys­tal formation using microwave-assisted co­crys­tallization (Ahuja *et al.*, 2020 ▸) was adopted using a Biotage Initiator+ and a 2∼5 ml reaction vial. Most phases isolable by solvothermal or LAG methods could be prepared phase pure in a fast and convenient manner in high yield (80–90%) on a 1 mmol scale in 2 ml 1-butanol (20 min heating at 140 °C and 20 min cooling).

### X-ray crystallography

Crystal data, data collection and structure refinement details are summarized in Table 1 ▸. Organic H atoms were placed geometrically and treated with riding constraints and displacement parameters derived from the C atoms to which they were attached. All CH and CH_2_ groups had *U*_iso_(H) values fixed at 1.2 times the *U*_eq_ of the parent C atom. Methyl groups were idealized as freely rotating CH_3_ groups and *U*_iso_(H) values were fixed at 1.5 times the *U*_eq_ of the parent C atom. H atoms on N atoms of the Tph alkaloids and H atoms on O atoms of the flavonoids were located in dif­ference maps and refined with individual isotropic displacement parameters. Disorder, for example in the case of racemic hesperetin and di­hydro­myricetin, was handled using *OLEX2* by defining separate parts, which assists maintaining separate geometry, appropriate bonding connectivity and riding H atoms (Nisar *et al.*, 2018 ▸).

## Results and discussion

### Background – alkaloid–flavonoid co­crys­tal formation

A search of the Cambridge Structural Database (CSD; Groom *et al.*, 2016 ▸) reveals that the structures of a considerable number of co­crys­tal phases between common alkaloid and flavonoid mol­ecules have been reported. Most of the previous studies have concentrated on the xanthine alkaloids, such as caffeine (Trask *et al.*, 2005 ▸) and its de­methyl­ated analogues theobromine (Sanphui & Nangia, 2014 ▸) and theo­phyl­line (Trask *et al.*, 2006 ▸). A variety of crystallization methodologies were adopted in these various studies (Karimi-Jafari *et al.*, 2018 ▸; Sakhiya & Borkhataria, 2024 ▸). A report on the use of caffeine co­crys­tals to effect a ‘green technology’ separation of a mixture of common flavonoids, namely, myricetin, quercetin and baicalein [see Figs. 1(*b*)–(*d*) ▸], based on differential solubility of the co­crys­tals formed, was quite noteworthy (Xia *et al.*, 2021 ▸).

**Figure 2:**
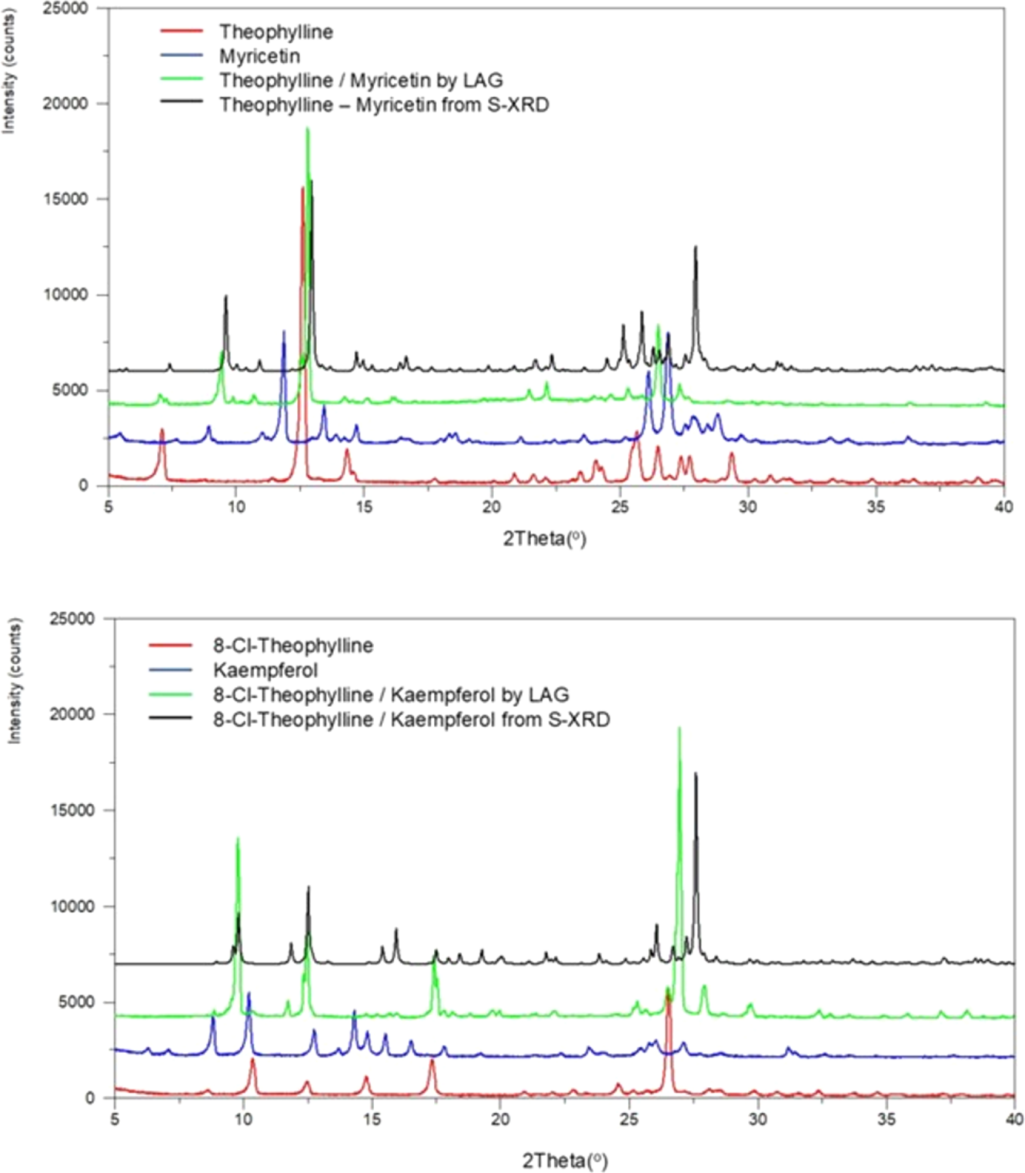
Powder X-ray diffractograms for alkaloid–flavonoid mol­ecular pairs. Examples from the theo­phyl­line–myricetin (**1c**; upper) and 8-chloro­theo­phyl­line–kaempferol (**2a**, lower) systems.

We sought to investigate this issue further with a more general investigation of alkaloid–flavonoid co­crys­tal formation using two steps. The first is an LAG screening through co-grinding of starting materials, using a small amount of solvent to help the process. The polar protic solvent methanol is suitable as a common ‘sparing’ solvent for many com­pounds in both the alkaloid and the flavonoid families. Typically, grinding over a period of 3 h is then sufficient to determine whether any change will occur under these conditions and the resulting powders can be inspected by powder X-ray diffraction (PXRD). Examples of successful co­crys­tal formation are given in Fig. 2 ▸ for the mol­ecular pairs theo­phyl­line–myricetin (**1c**, Tph–Myr) and 8-chloro­theo­phyl­line–kaempferol (**2a**, 8-Cl-Tph–Kmp).

In cases where a clear PXRD change is seen, follow-up crystallizations using a heating–cooling co-precipitation ap­proach were undertaken. A mixed aqueous methano­lic solution was heated solvothermally for several hours to effect full dissolution of the co-formers and then cooled slowly to afford co­crys­tal formation, typically in agreement with LAG-prepared phases with specimens of suitable size for crystal structure determination. We have carried out this process in around 250 alkaloid–flavonoid binary combinations, resulting in the isolation of 75 co­crys­tal phases (Ye, 2024 ▸). These results indicate a strong tendency for co­crys­tal formation between the two mol­ecular families. This is readily ascribed to the pro­pen­sity for more optimal hy­dro­gen-bonding arrangements in the co­crys­tal phases, with alkaloids being rich in hy­dro­gen-bond-acceptor moieties and flavonoids being rich in hy­dro­gen-bond-donor groups. This was borne out by the variable tendency to co­crys­tal formation based on the degree to which these statements applied to individual mol­ecules. Notwithstanding, all alkaloid and flavonoid mol­ecules studied were found to form at least one co­crys­tal phase (Ye, 2024 ▸).

In order to check the packing efficiencies for co­crys­tals *versus* their co-formers, we undertook the structure determination of all co-formers studied at a common tem­per­a­ture of 100 K. In many cases, the structures for these common com­pounds are well established and, in some cases, several polymorphic forms have been reported. We were surprised, however, that 8-halotheo­phyl­lines (*X* = Br, Cl) were unreported and hence carried out new structure determinations for these (Ye *et al.*, 2025 ▸). Several new polymorphs were uncovered for these, which were all distinct from those for the parent mol­ecule theo­phyl­line despite the perturbation of just one atom in the mol­ecule (Fig. 1 ▸). Our density functional theory (DFT) calculations supported the notion that these structural changes came from lowering the basicity of the N9 atom. Electrostatic potential (ESP) calculations indicate a lowering of charge on N9 from −0.62 in Tph itself to −0.52 in 8-Cl-Tph and to −0.46 in 8-Br-Tph (Ye *et al.*, 2025 ▸). This supports the observed switch from N7—H⋯N9 hy­dro­gen-bonded polymorphs for theo­phyl­line (Tph) to N7—H⋯O hy­dro­gen-bonded polymorphs for 8-*X*-Tph. We speculated that this change might affect co­crys­tal formation of 8-*X*-Tph with flavonoids as well.

The powder X-ray diffractograms from LAG of stoichiometric ratios of the alkaloid and flavonoid were measured and com­pared with the starting reagents and the simulated patterns of the isolated and structurally characterized co­crys­tals. Given sufficient grind time, product patterns matched well to those later simulated from single-crystal structure determinations, after taking into account the tem­per­a­tures of measurement (Fig. 2 ▸).

### Cocrystals of theo­phyl­line (Tph) and flavonoids

Three co­crys­tal phases of theo­phyl­line (Tph) have been reported previously (Wang *et al.*, 2022 ▸; Zhu *et al.*, 2017 ▸; Sun *et al.*, 2023 ▸). We were able to prepare all such phases in a reasonably phase-pure manner, by use of both LAG and our heating–cooling co-precipitation process. In all, around 15 com­mon flavonoids were screened for co­crys­tal formation with Tph. A total of six co­crys­tal phases were isolated using the flavonoids depicted in Fig. 1 ▸, namely, kaempferol (**a**), quer­cetin (**b**), myricetin (**c**), baicalein (**d**), hesperetin (**e**) and di­hydro­myricetin (**f**), which are listed in Table 2 ▸. The previously reported phases (CSD refcodes JATPIH, KAM­QIB and NEXSIW) will not be discussed in further detail here and references are given in Table 2 ▸. It may be noted that the phases may be of 1:1 or 2:1 stoichiometry, and often hydrated. NEXSIW is an aceto­nitrile solvate. Use of MeOH and our ‘standard’ method of co­crys­tallization yielded no other co­crys­tal phase in this system. Crystal data summaries at 100 K for the three new theo­phyl­line–flavonoid co­crys­tal phases **1a**, **1c** and **1e** are given in Table 1 ▸.

**Figure 3:**
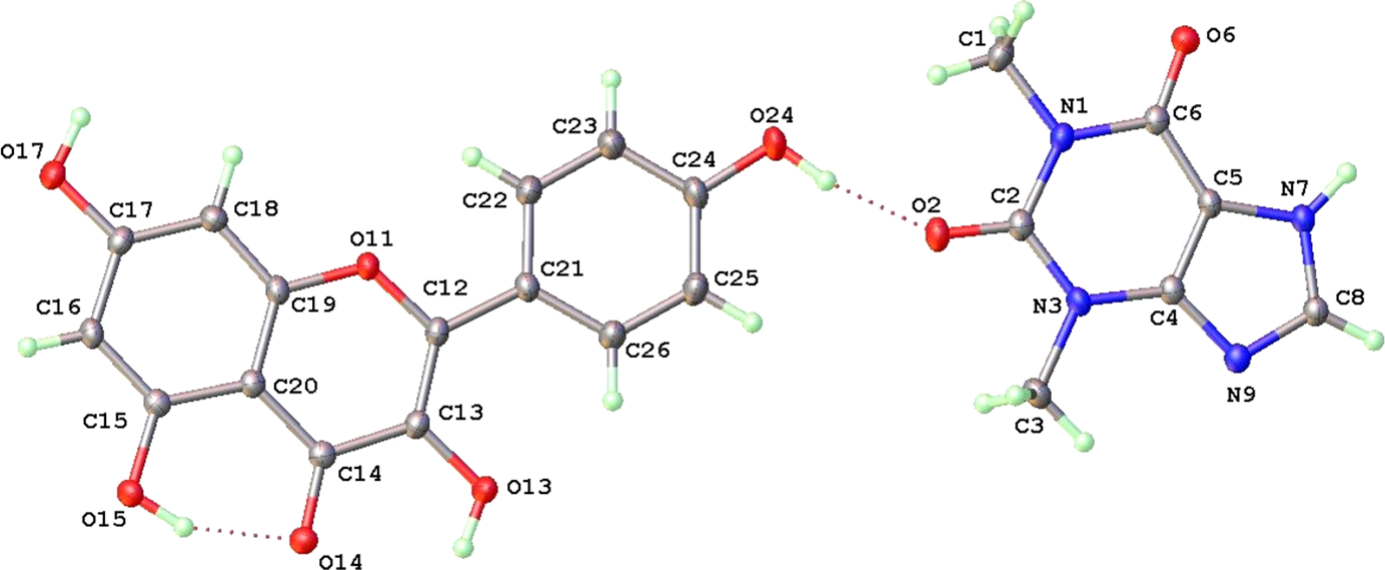
The mol­ecular pair for theo­phyl­line–kaempferol (**1a**), showing the atom-labelling scheme (50% probability displacement ellipsoids).

**Figure 4:**
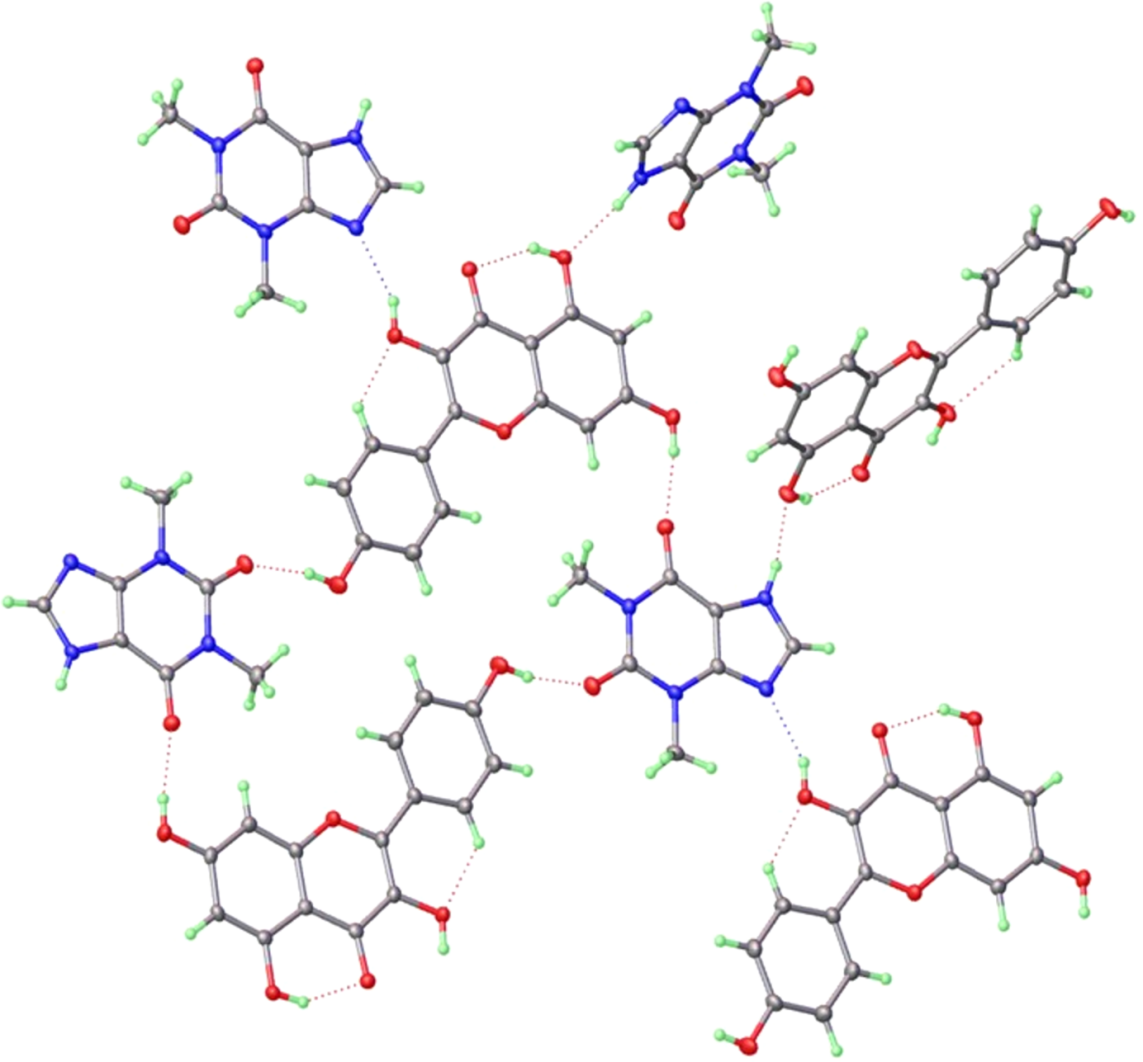
The packing environment for theo­phyl­line–kaempferol (**1a**), showing the alkaloid as net hy­dro­gen-bond acceptor and the flavonoid as net hy­dro­gen-bond donor.

Our first unreported co­crys­tal phase from theo­phyl­line is produced with kaempferol and an unsolvated 1:1 phase is formed, so the asymmetric unit is a mol­ecular pair, shown in Fig. 3 ▸. The phase Tph–Kmp (**1a**) forms a relatively com­plex packing, that results in the net hy­dro­gen-bonding inter­actions to neighbours shown in Fig. 4 ▸. Each theo­phyl­line is hy­dro­gen bonded to four kaempferol neighbours and *vice versa*. Three hy­dro­gen bonds are donated by the flavonoid and one by the alkaloid, so there is *net hy­dro­gen bonding* from one entity to the other, as postulated in our general concept of why these mol­ecular classes should have a tendency to co­crys­tal formation with each other.

**Figure 5:**
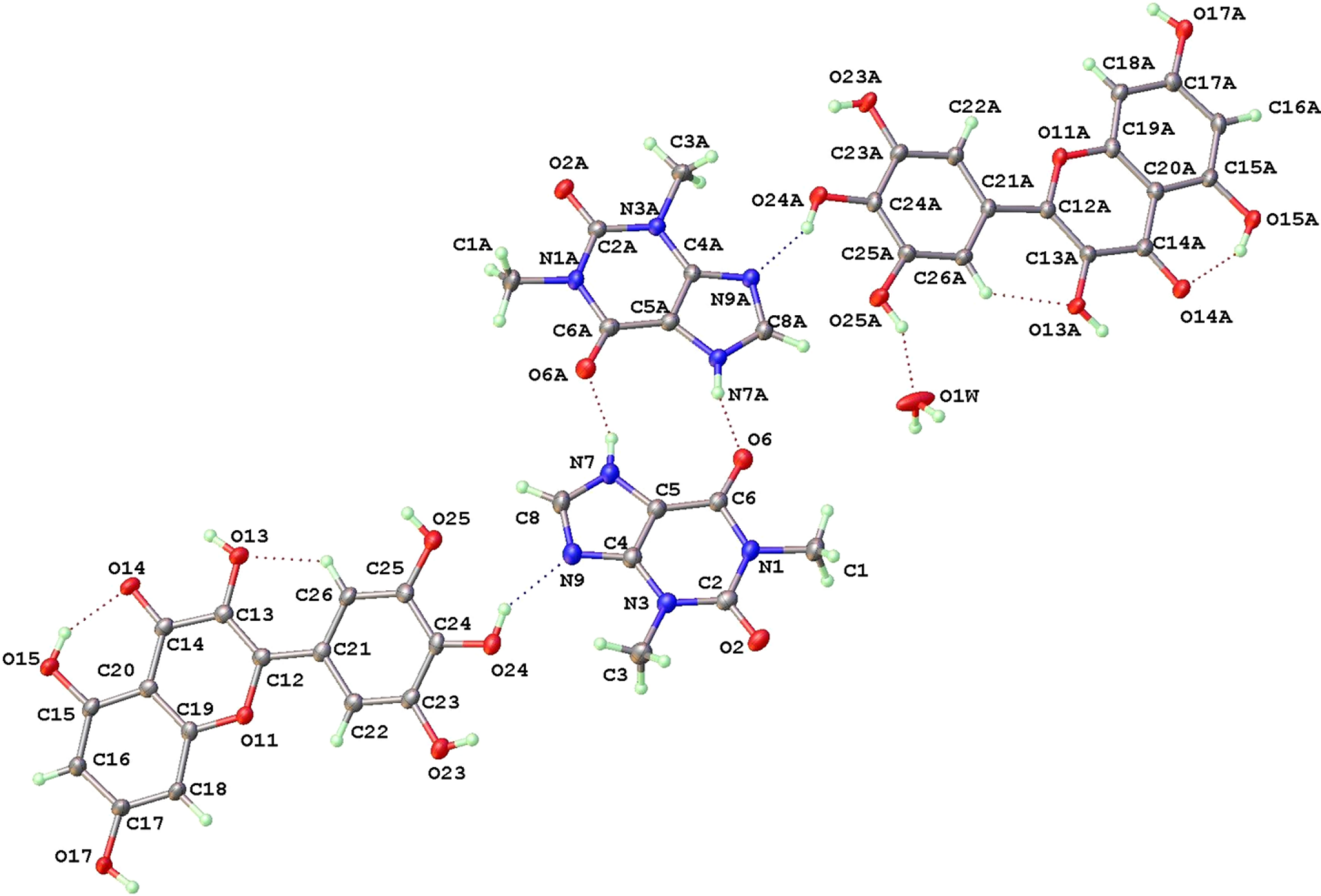
The asymmetric unit for theo­phyl­line–myricetin hemihydrate (**1c**), showing the atom-labelling scheme (50% probability displacement ellipsoids).

Of the other newly prepared co­crys­tal phases, Tph–myricetin is a hemihydrate that crystallizes in the space group *P*2_1_/*c*. The asymmetric unit com­prises two theo­phyl­line and two myricetin mol­ecules and a water mol­ecule (Fig. 5 ▸) The structure is notable in that the two independent Tph mol­ecules form a hy­dro­gen-bonded dimer *via* two N7—H⋯O6′ hy­dro­gen bonds that create an 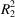
(10) ring (for this topological notation of hy­dro­gen bonds, see Etter *et al.*, 1990 ▸).

**Figure 6:**
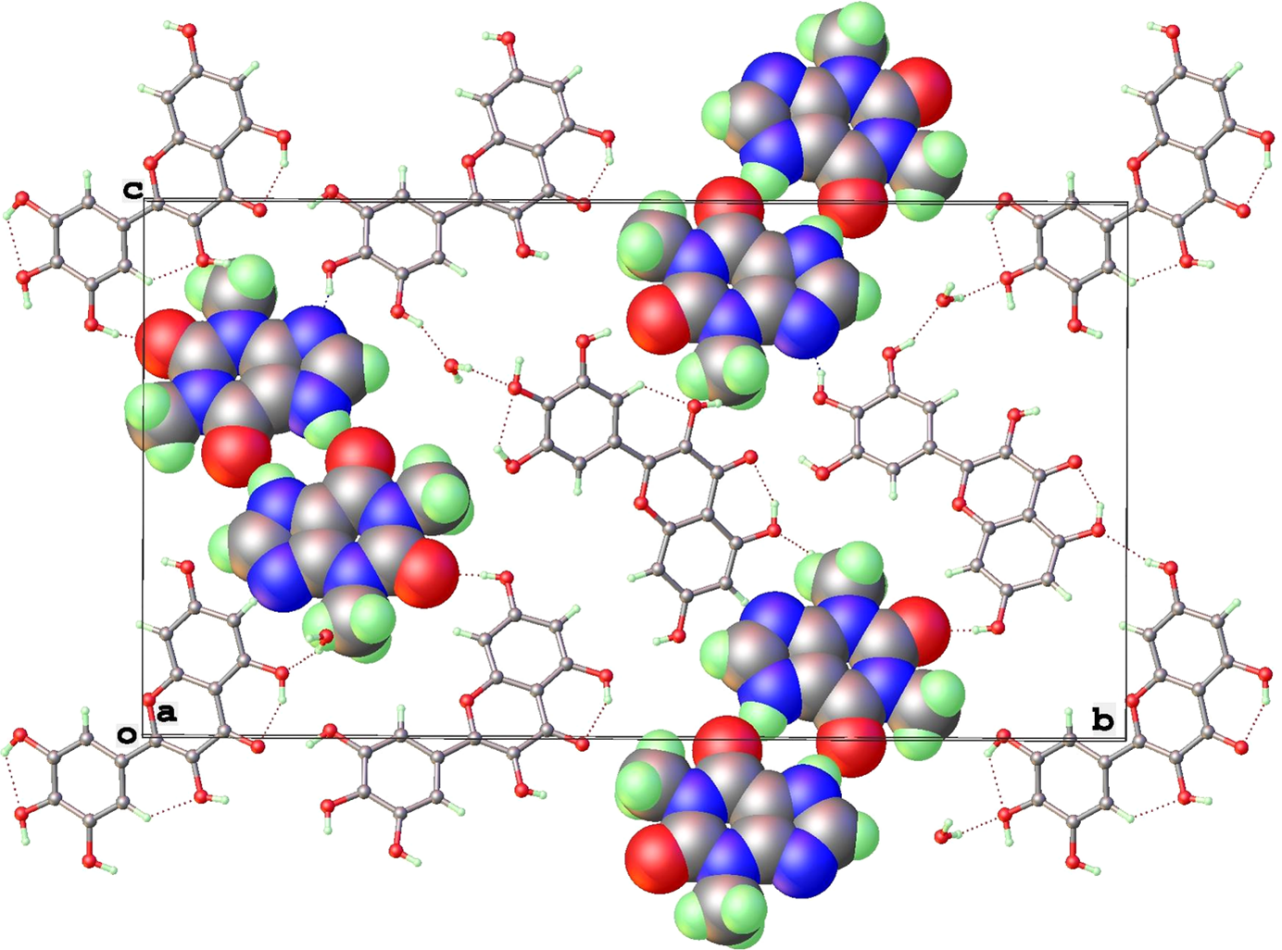
Packing diagram for theo­phyl­line–myricetin hemihydrate (**1c**), viewed along the *a* axis.

In the various polymorphs of theo­phyl­line itself, this is not thermodynamically favoured, though it is found in one metastable polymorph (Dyulgerov *et al.*, 2015 ▸). When viewed along the shorter 7.3 Å *a* axis, isolated mol­ecular stacks of Tph dimers (space-filled) are seen surrounded by six myricetin stacks (Fig. 6 ▸). Overall each Tph dimer pair accepts four hy­dro­gen bonds from phenolic groups on the two myricetins. The water acts as both a hy­dro­gen-bond donor and acceptor to the two myricetin mol­ecules and breaks the pseudosymmetry that can be seen within the asymmetric unit (Fig. 5 ▸).

**Figure 7:**
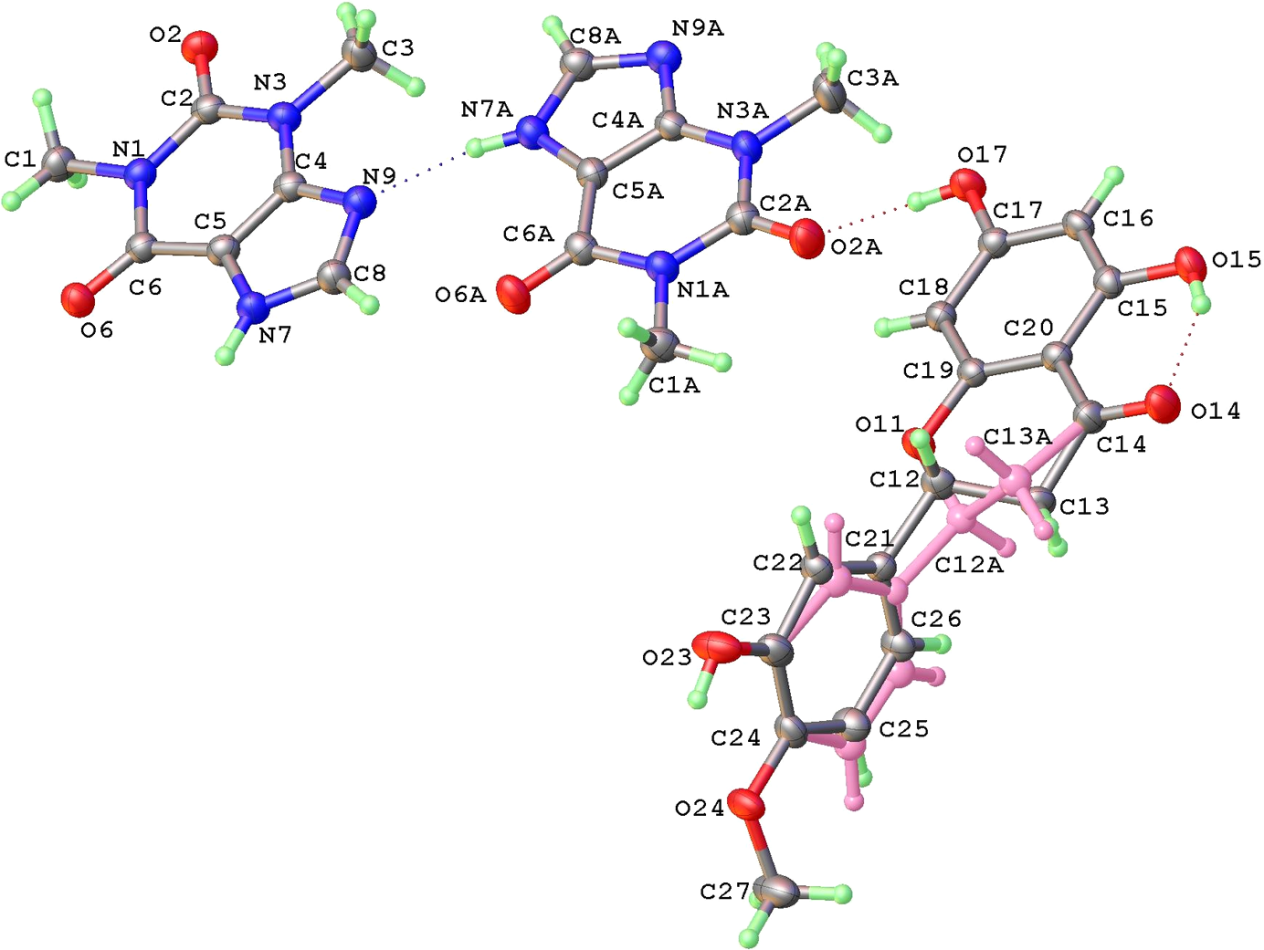
The asymmetric unit for theo­phyl­line–*rac*-hesperetin 2:1 co­crys­tal (**1e**), with the solvent excluded. The minor disordered com­ponent (30%) is shown in pink.

**Figure 8:**
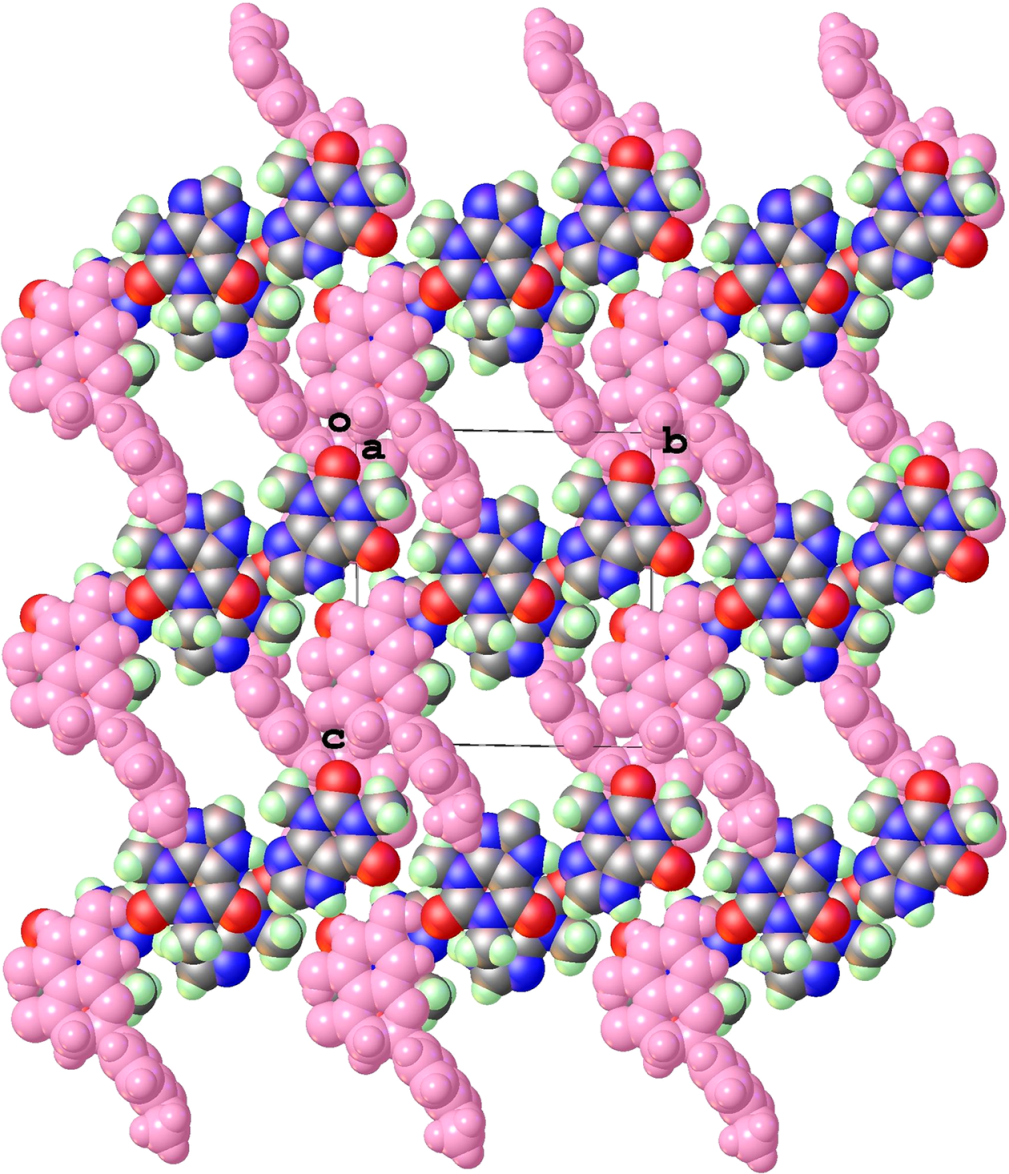
Space-filling diagram of the theo­phyl­line–*rac*-hesperetin 2:1 co­crys­tal (**1e**), viewed along the *a* axis, showing large open solvent channels. The hesperetin mol­ecules are shaded in pink.

Finally, one further co­crys­tal phase that we have isolated from theo­phyl­line and flavonoids is the solvated 2:1 co­crys­tal theo­phyl­line–*rac*-hesperetin (**1e**). Hesperetin is a flavonoid with a single chiral centre and is found naturally as hesperidin, a rutinoside (6-*O*-α-l-rhamnosyl-d-glucoside), in which the chirality is 2*S* (Pryzynska, 2022 ▸). However, following isolation, it is usually offered commercially as a racemate. In this case, racemic hesperetin was co­crys­tallized with theo­phyl­line. The product phase crystallizes in the triclinic space group *P*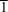 with a 2:1 ratio of alkaloid to flavonoid and a roughly 70:30 disorder of the racemic hesperetin at a single crystallographic site. In the asymmetric unit shown in Fig. 7 ▸, the chiral centre is 70%*R* C12 and 30%*S* C12*A*. The opposite enanti­omeric ratio will be found at the inverted site of the unit cell. Attempted refinement in chiral *P*1 space group failed to improve the modelling indicating there is no spontaneous resolution of the *rac*-hesperetin in this co­crys­tal phase. The phase is highly solvated and, when viewed along the short 6.8 Å *a* axis, the structure reveals large channel voids of roughly 7 × 10 Å dimension (Fig. 8 ▸). The application of SQUEEZE (Spek, 2015 ▸) indicates around 37 e^−^ for a contiguous accessible void of around 112 Å per asymmetric unit. The disordered solvent is com­patible with a combination of two waters and one methanol mol­ecule.

### Flavonoid cocrystal formation for 8-chloro and 8-bromo­theo­phyl­line

No structural reports existed for 8-chloro- or 8-bromo­theo­phyl­line polymorphs or co­crys­tals in the CSD. However, the well-known drug dimenhydrinate (Dramamine) is the diphenhydramine salt of the 8-Cl-Tph anion, which was structurally determined (Putra *et al.*, 2016 ▸). Recently, we have reported the polymorphic forms of 8-Cl-Tph and 8-Br-Tph (Ye *et al.*, 2025 ▸). Inter­estingly, these structures exclusively favoured N—H⋯O hy­dro­gen bonding between the alkaloid mol­ecules, that we believed was due to a reduction in N9 basicity. Since the introduction of the 8-halo substituent affected the structural polymorphism of these theo­phyl­line derivatives, we also wanted to see the potential effect on co­crys­tal formation. Accordingly, the same 15 flavonoids were screened for co­crys­tal formation with 8-*X*-Tph as for Tph. This time the only flavonoids affording co­crys­tals with 8-*X*-Tph were kaempferol (**a**) and myricetin (**c**). The phases produced were found by PXRD to be isostructural between the 8-Cl-Tph and 8-Br-Tph cases. The phase details are given in Table 3 ▸ and crystal data summaries in Table 1 ▸. Attempts to use microwave crystallization to afford these phases in a convenient manner from methanol also yielded a further solvated co­crys­tal phase for 8-Br-Tph and kaempferol (**3a′**). The sol­vated co­crys­tal formation can, however, be suppressed using 1-butanol (140 °C) in the microwave-assisted co­crys­tallization process from which pure **3a** can be isolated.

**Figure 9:**
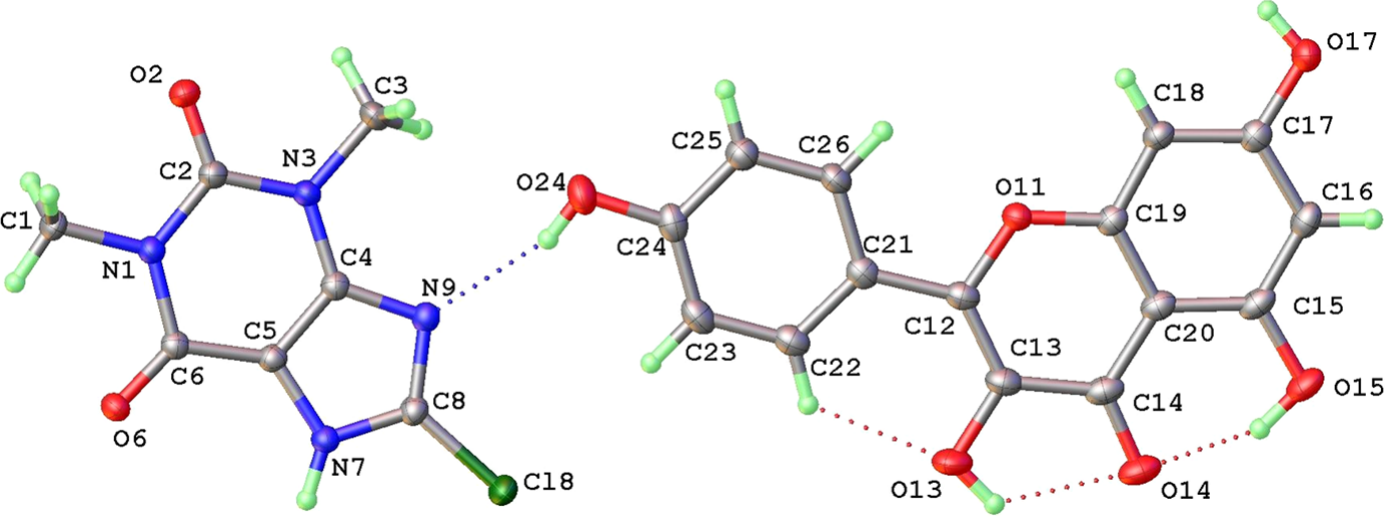
The labelling scheme for the 8-Cl-theo­phyl­line–kaempferol 1:1 co­crys­tal (**2a**), similarly adopted by the isostructural 8-Br analogue **3a**.

**Figure 10:**
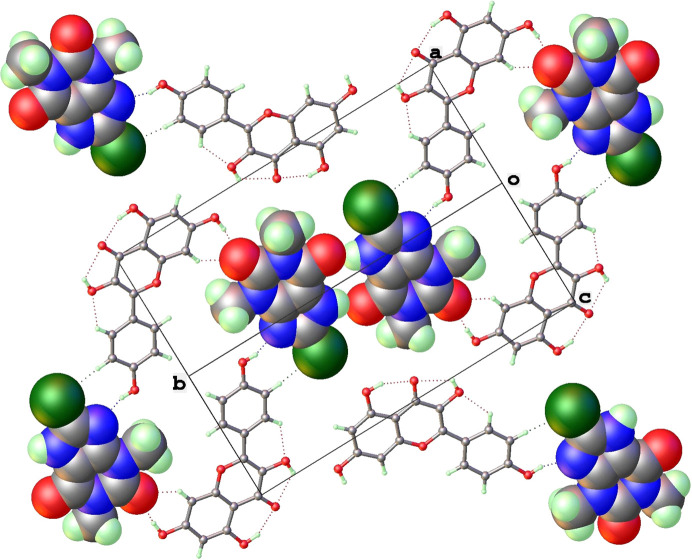
Packing diagram for the 8-Cl-theo­phyl­line–kaempferol 1:1 co­crys­tal (**2a**), viewed along [101] and showing 8-Cl-Tph dimers (space-filled mol­ecules).

Phases **2a** and **3a** are isostructural, and the asymmetric unit is a simple mol­ecular pair, as shown in Fig. 9 ▸. In all these co­crys­tals, the halotheo­phyl­lines form mol­ecular dimers through N7—H⋯O6 hy­dro­gen bonds, as described above for **1c**, and this can be seen in the packing diagram for **2a** shown in Fig. 10 ▸. The remaining keto O2 atom then typically serves as an acceptor of a strong hy­dro­gen bond from a flavonoid OH group, whilst N9 is involved either in a weak hy­dro­gen bond, as seen in Fig. 9 ▸ (N9—H⋯O24 = 2.87 Å), or not at all. In **2a** and **3a**, layer structures are found in which the 8-*X*-Tph dimers are surrounded by six neighbouring kaempferol mol­ecules (see Fig. 10 ▸).

**Figure 11:**
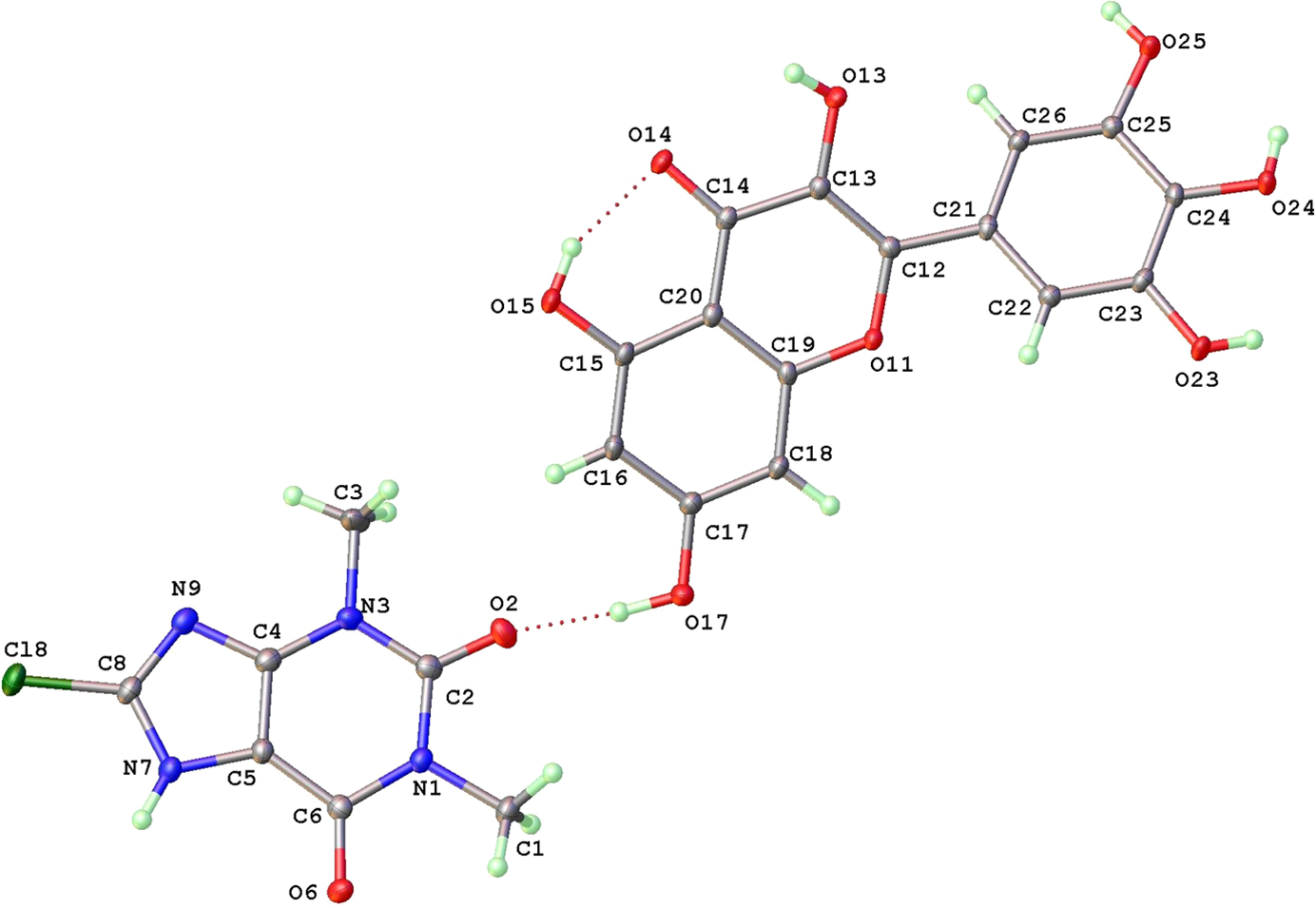
The labelling scheme for the 8-Cl-theophilline–myricetin 1:1 co­crys­tal (**2c**), similarly adopted by the isostructural 8-Br analogue **3c**.

**Figure 12:**
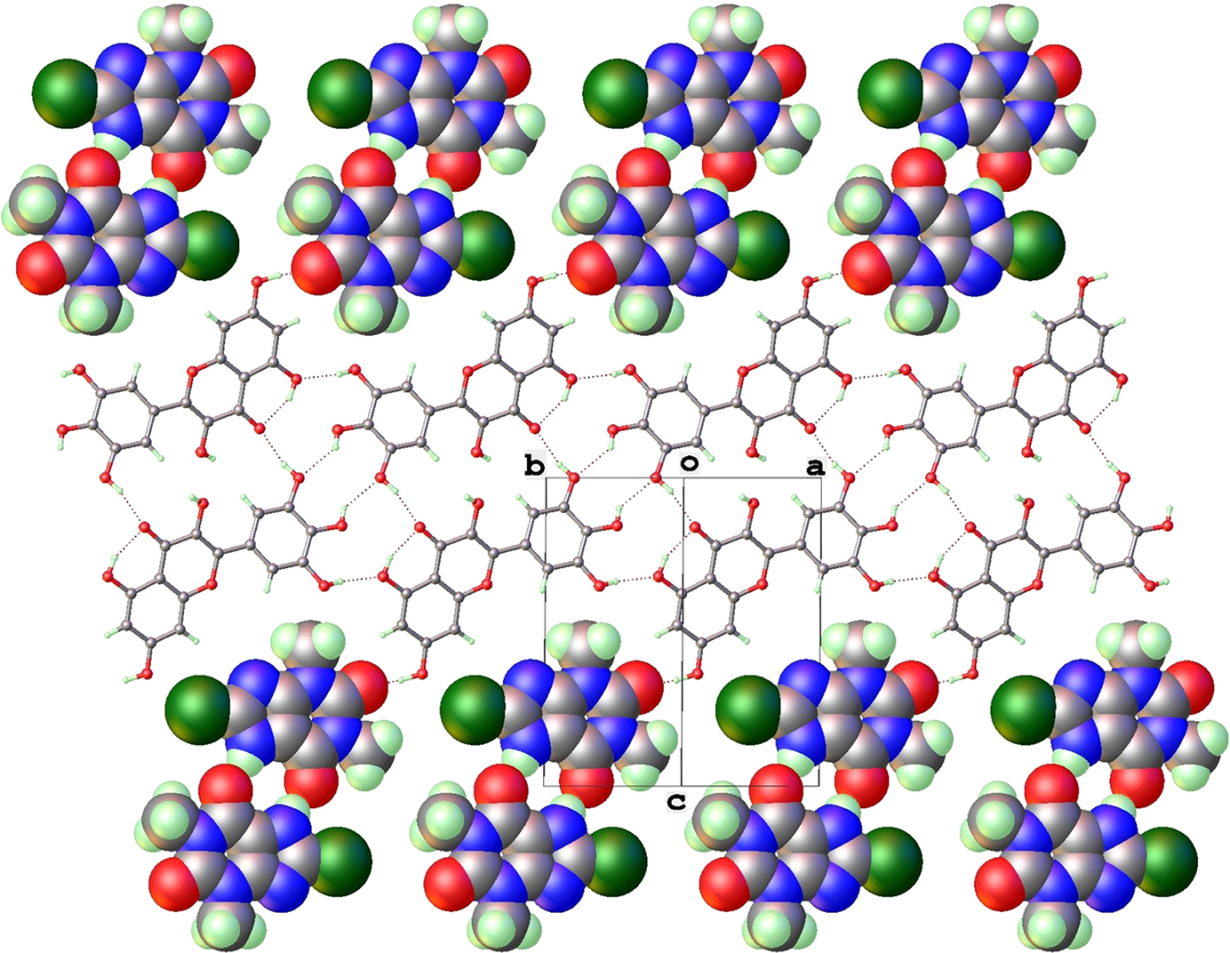
Packing diagram for **2c**, viewed along [110], showing the segregation of myricetin double-stranded chains and 8-Cl-Tph dimers (space-filled).

The co­crys­tals of 8-*X*-Tph with myricetin, **2c** (*X* = Cl) and **3c** (*X* = Br), are also an isostructural pair with simple halogen inter­change. The asymmetric unit and atom-labelling scheme for **2c** are shown in Fig. 11 ▸. These phases also form layer structures, but in **2c** and **3c**, there is segregation of the 8-*X*-Tph dimers and myricetin mol­ecules within the layers, as shown in the packing diagram in Fig. 12 ▸.

**Figure 13:**
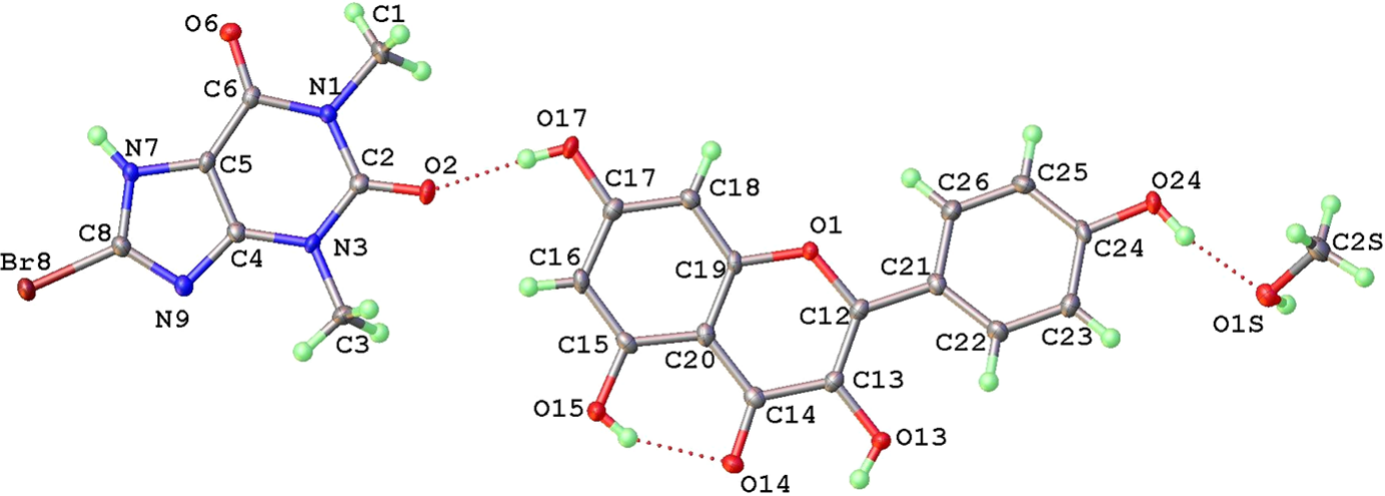
The asymmetric unit and labelling scheme for the 8-Br-theo­phyl­line–kaempferol–MeOH 1:1:1 co­crys­tal (**3a′**).

**Figure 14:**
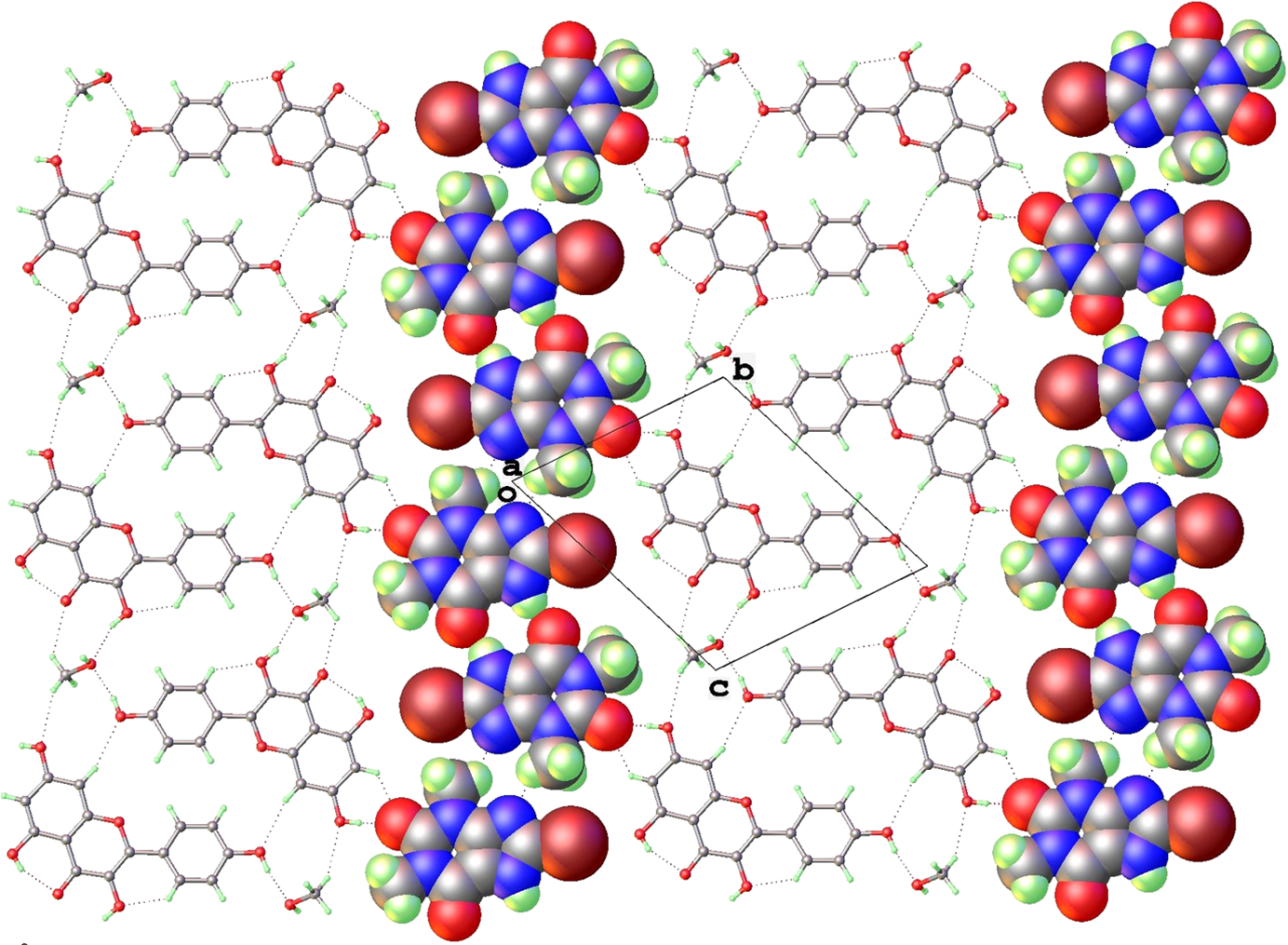
Packing diagram for the 8-Br-theo­phyl­line–kaempferol–MeOH 1:1:1 co­crys­tal (**3a′**), viewed along [100], showing alternating strands of 8-Br-Tph dimers and solvated kaempferol mol­ecules.

Finally, a further methano­lated co­crys­tal phase **3a′** was isolated for the 8-Br-Tph–Kmp system, upon microwave-assisted synthesis in MeOH, that was not found previously by LAG or solvothermal crystallization. The asymmetric unit and atom-labelling scheme is shown in Fig. 13 ▸. Unlike phase **3a**, the alkaloid dimers are not isolated but also segregate from solvated kaempferol in one-dimensional strands (Fig. 14 ▸). This phase type may be favoured through the formation of an attractive Br⋯O inter­action of 3.024 (2) Å.

### Structures of coformers: specific volume com­parison for co­crys­tals

**Figure 15:**
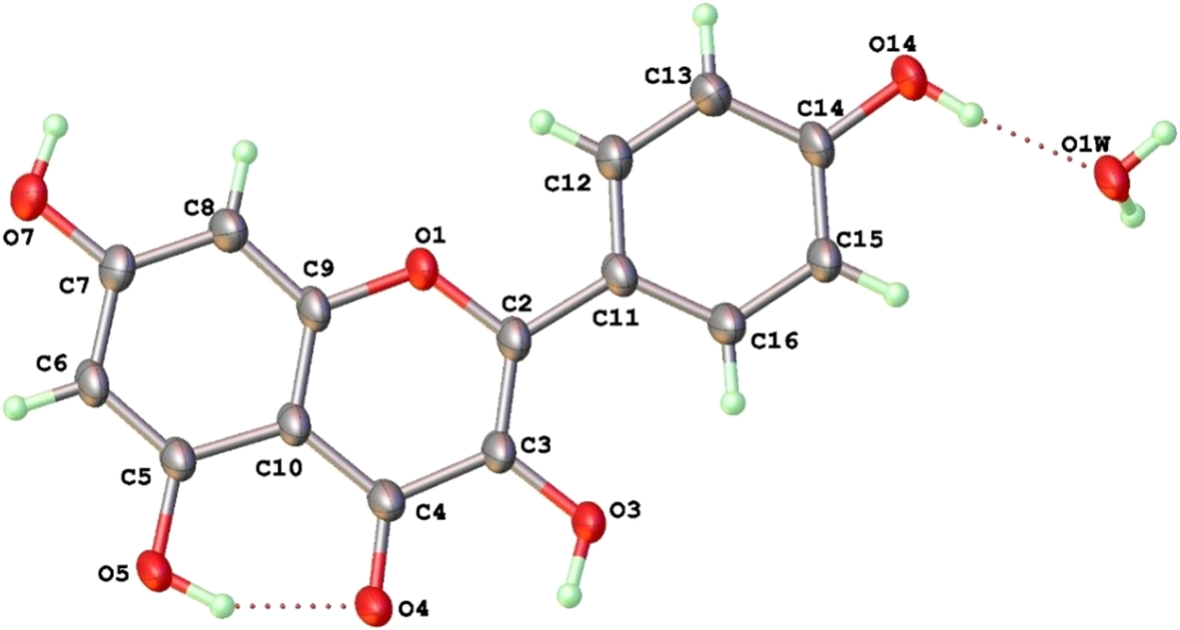
The asymmetric unit and atom-labelling scheme for kaempferol hydrate (**a·H_2_O**) (50% probability displacement ellipsoids).

The crystal structures at 100 K of theo­phyl­line (Fucke *et al.*, 2012 ▸) and the 8-chloro and 8-bromo analogues 8-*X*-Tph were reported recently by us (Ye *et al.*, 2025 ▸). Several crystal structures of the various flavonoid coformers were reported previously by others, many of them as hydrated or solvated phases; where possible, we have redetermined these at 100 K. Inter­estingly, no structural entry existed for kaempferol. We have grown this as a monohydrate (**a·H_2_O**) through the ambient evaporation of an acetonitrile solution and report its crystal structure here (Fig. 15 ▸). The structural details are given in Table 1 ▸.

Table 4(*a*) ▸ gives a summary of the alkaloid and flavonoid coformer structures (at or close to 100 K), along with the corresponding mol­ecular volume (*V*_mol_) for the alkaloid or flavonoid of inter­est. The variable volume of water or solvent mol­ecules make com­parison of specific mol­ecular volumes for co­crys­tals and coformers slightly more involved. However, a useful com­parison of packing efficiency may still be attempted and is provided for the co­crys­tals in Table 4(*b*) ▸. In general, it was anti­cipated that the specific volume for a co­crys­tal would be less than the sum of the individual coformers; however, surprisingly, this was not found to be the case for these co­crys­tals. If mol­ecular volumes of 20, 40 and 60 Å^3^ are assigned to water, methanol and aceto­nitrile, respectively, then the combined volumes of the alkaloid and flavonoid mol­ecules in the co­crys­tals are actually *equal to or greater than* those from the parent coformer crystals themselves.

### Rationalization of co­crys­tal formation

Historically, recrystallization of mol­ecular mixtures was used as a tool for purification, relying on the lower solubility of one mol­ecule than the others to initiate its precipitation. The advent of crystal engineering has developed ideas of mol­ecular recognition in organic crystal structures that can also be adapted to assist the intentional co­crys­tallization of mol­ecules, with driving forces for this based on inter­molecular recognition, supra­molecular synthon formation, electrostatic inter­actions or better hy­dro­gen bonding or packing efficiency. Our idea in this project was to survey the tendency of co­crys­tal formation between two extensive and fundamental classes of natural product, *i.e.* alkaloids, which typically have a surplus of hy­dro­gen-bond-acceptor functionalities (ring N or exocyclic keto O atoms), and flavonoids, which have a surplus of OH-donor functionalities to keto O-atom acceptors.

In examining the co­crys­tal formation for the anti­malarial com­pound 11-aza­ar­tem­is­in­in, we found that it could form co­crys­tals with around 50% of organic carb­oxy­lic acids studied (Nisar *et al.*, 2018 ▸). This appeared to be supported by a favourable supra­molecular heterosynthon between the acid and the amide. In the cases that were successful, the specific volumes of the co­crys­tal phases were typically less than that for the com­ponent co-formers in their own crystal. However, when salicylic acids were studied exclusively, the co­crys­tal formation probability was over 95%, due to strengthening of this heterosynthon, and a number of these co­crys­tals were less efficiently packed than their parent phases (Li *et al.*, 2022 ▸; Li, 2024 ▸). Thus, a sufficient improvement in hy­dro­gen bonding can overcome packing deficiency.

In a study of 350 organic co­crys­tals by DFT calculations, Taylor & Day (2018 ▸) found that the vast majority (>95%) were thermodynamically preferred and by an average of 8 kcal mol^−1^. However, several factors appear to be operating in tandem to lead to this stabilization. By combining two key descriptors: (i) packing efficiency and (ii) hy­dro­gen-bond strength; these gave good correlations with the stabilization of the co­crys­tal phases. In particular, those phases with lower packing efficiency typically had optimization of hy­dro­gen bonds in the co­crys­tal phases to offset this.

Our surprising findings that the theo­phyl­line–flavonoid co­crys­tals in these studies typically have *less efficient* packing than the parent coformer phases led us to next examine hy­dro­gen bonding and whether this was optimized in the co­crys­tal phases that we have isolated (Table 5 ▸). We present an analysis of Tph and 8-*X*-Tph with kaempferol and myricetin, since these phases are less solvated than some of the other systems and the structural effect of the 8-halo substituent can also be examined. In general, alkaloid–flavonoid co­crys­tallization does seem to be favoured due to net hy­dro­gen-bond formation from the H-atom-donor-rich flavonoid to the H-atom-acceptor-rich alkaloid.

In principle, theo­phyl­line has three good acceptors, *i.e.* N9, O2 and O6. In co­crys­tal phase **1a** (Tph–Kmp), there are three hy­dro­gen bonds from the flavonoid to these three acceptors, including a short O24—H⋯O2 inter­action (2.68 Å), as well as a ‘reverse’ hy­dro­gen bond from the theo­phyl­line N7—H group to the flavonoid. In the other Tph co­crys­tal phases **1c** (Tph–Myr·0.5H_2_O) and **1e** (Tph–Hes 2:1 solvate), once more a surplus of two hy­dro­gen bonds are donated from the flavonoid to the alkaloid and the N9 atom is involved as acceptor. The previously reported co­crys­tals of theo­phyl­line with flavonoids show generally similar findings (Wang *et al.*, 2022 ▸; Zhu *et al.*, 2017 ▸; Sun *et al.*, 2023 ▸).

In examining the structures of theo­phyl­line mol­ecular crystals themselves, we found that the N9 position is deactivated as an acceptor upon substitution of the 8-*X* groups Cl and Br (Ye *et al.* 2025 ▸). This deactivation may lie at the heart of why fewer co­crys­tal phases are formed for the 8-halotheo­phyl­lines. This is supported by the observation that in those co­crys­tal types that are found for 8-*X*-Tph and flavonoids, the N9 group is either in just a weak hy­dro­gen bond (OH⋯N9 = 2.87 Å for **2a** and OH⋯N9 = 2.88 Å for **3a**) or is not involved at all (phases **2c**, **3c** and **3a′**).

It may be noted that among the flavonoids screened for co­crys­tallization, kaempferol (Kmp) and myricetin (Myr) are the most active coformers, with 12 co­crys­tals each from 15 common alkaloids. This may be explained, in part, by the fact that the com­pounds themselves are difficult to crystallize in the anhydrous form, so that pure coformer crystals are uncom­petitive solid phases. Both kaempferol (reported here) and myricetin (Ren *et al.*, 2019 ▸) form monohydrates, but so does quercetin (Domagała *et al.*, 2011 ▸). Analysis of the respective mol­ecular volumes (Table 4 ▸) indicates that quercetin monohydrate is more efficiently packed. Hence, quercetin has a slightly less pronounced tendency to co­crys­tal formation, with eight co­crys­tals, a number of co­crys­tal phases that is similar to baicalein which has a phenyl group.

The 8-halo-Tph phases **2a**/**3a** and **2c**/**3c** are isostructural pairs; however, efforts to isolate the 8-Cl-Tph analogue of the methanol-solvated phase 8-Br-Tph–Kmp·MeOH (**3a′**) were unsuccessful. Examination of the **3a′** structure shows that a favourable Br⋯O15 contact of 3.024 (2) Å for the halogen-bond inter­action exists in this phase. This would be considerably weaker if Cl is substituted for Br, offering an explanation of why the analogous isostructural methanol solvate **2a′** is not formed.

In the cases pre­sent­ed here, the thermodynamic stability of the majority of the co­crys­tal phases relative to their com­ponents was established through their formation *via* direct LAG of the coformers in the appropriate stoichiometric ratio (1:1 or 2:1). PXRD indicated that almost all co­crys­tal phases for theo­phyl­line could be formed, with the exception of di­hydro­myricetin, which is an aceto­nitrile solvate, and hesperetin, which forms a highly solvated co­crys­tal phase, which we believe is an unstable kinetic product.

Where LAG is successful, the use of microwave-assisted co­crys­tallization (Ahuja *et al.*, 2020 ▸) appears to be the most convenient ap­proach to form stable phases in good yield and purity, as given for eight phases in Table 6 ▸. Yields of 80–90% were obtained for a typical 1 mmol scale in 2 ml 1-butanol, applying 20 min heating at 140 °C, followed by a 20 min cooling cycle, with the solids centrifuged, filtered and dried. When LAG is applied directly, some slight contamination of the co­crys­tals with starting phases is hard to eliminate entirely.

## Conclusions

Screening for co­crys­tal formation of 13 common flavonoids with a family of alkaloids that are electronically perturbed, shows that theo­phyl­line (Tph) has a markedly stronger tendency for co­crys­tallization com­pared to its 8-halo analogues. A total of six flavonoid co­crys­tals were isolated for Tph, three of which were reported previously, whilst only two flavonoids work for 8-Cl-Tph or 8-Br-Tph. Principally, these are the unsolvated 1:1 phases with kaempferol (**2a**/**3a**) and myricetin (**2c**/**3c**), which are isostructural for the 8-Cl or 8-Br substituents. *None* of the successful co­crys­tallization outcomes of flavonoids with any of these theo­phyl­line alkaloids appear to be driven by improved packing efficiency. The marked dif­ference in co­crys­tal formation tendency for Tph com­pared to its halo derivatives may be influenced by the deactivation of the alkaloid N9 atom as a hy­dro­gen-bond acceptor in the halogenated mol­ecules. In general, the co­crys­tal phases reported here are thermodynamically com­petitive and can be prepared by co-precipitation from cooling hot solutions (solvothermal), by LAG of co-formers or, most conveniently, through microwave-assisted co­crys­tallization from 1-butanol.

## Supplementary Material

Crystal structure: contains datablock(s) 1a-ywj135Cu100k_auto, 1c-ywj16bcult_auto, 1f-ywj137acu100k_auto, 2a-ywj65cult100k_auto, 2c-ywj68cult100k_auto, 3a_ywj75cult100k_auto, 3c-ywj230_cu100k_auto, 3a_prime_ywj75e_mo100k_auto, aH2O-ywj219_ga100k, global. DOI: 10.1107/S2053229626006637/oj3040sup1.cif

Structure factors: contains datablock(s) 1a-ywj135Cu100k_auto. DOI: 10.1107/S2053229626006637/oj30401a-ywj135Cu100k_autosup2.hkl

Structure factors: contains datablock(s) 1c-ywj16bcult_auto. DOI: 10.1107/S2053229626006637/oj30401c-ywj16bcult_autosup3.hkl

Structure factors: contains datablock(s) 1f-ywj137acu100k_auto. DOI: 10.1107/S2053229626006637/oj30401f-ywj137acu100k_autosup4.hkl

Structure factors: contains datablock(s) 2a-ywj65cult100k_auto. DOI: 10.1107/S2053229626006637/oj30402a-ywj65cult100k_autosup5.hkl

Structure factors: contains datablock(s) 2c-ywj68cult100k_auto. DOI: 10.1107/S2053229626006637/oj30402c-ywj68cult100k_autosup6.hkl

Structure factors: contains datablock(s) ywj75cult100k_auto. DOI: 10.1107/S2053229626006637/oj30403a-ywj75cut100k_autosup7.hkl

Structure factors: contains datablock(s) 3c-ywj230_cu100k_auto. DOI: 10.1107/S2053229626006637/oj30403c-ywj230_cu100k_autosup8.hkl

Structure factors: contains datablock(s) 3a_prime_ywj75e_mo100k_auto. DOI: 10.1107/S2053229626006637/oj30403a_prime_ywj75e_mo100k_autosup9.hkl

Structure factors: contains datablock(s) aH2O-ywj219_ga100k. DOI: 10.1107/S2053229626006637/oj3040aH2O-ywj219_ga100ksup10.hkl

Supporting information file. DOI: 10.1107/S2053229626006637/oj30401a-ywj135Cu100k_autosup9.cml

Supporting information file. DOI: 10.1107/S2053229626006637/oj30401c-ywj16bcult_autosup10.cml

Supporting information file. DOI: 10.1107/S2053229626006637/oj30401f-ywj137acu100k_autosup11.cml

Supporting information file. DOI: 10.1107/S2053229626006637/oj30402a-ywj65cult100k_autosup12.cml

Supporting information file. DOI: 10.1107/S2053229626006637/oj30402c-ywj68cult100k_autosup13.cml

Supporting information file. DOI: 10.1107/S2053229626006637/oj30403a_ywj75cult100k_autosup14.cml

Supporting information file. DOI: 10.1107/S2053229626006637/oj30403c-ywj230_cu100k_autosup15.cml

Supporting information file. DOI: 10.1107/S2053229626006637/oj30403a_prime_ywj75e_mo100k_autosup16.cml

Supporting information file. DOI: 10.1107/S2053229626006637/oj3040aH2O-ywj219_ga100ksup17.cml

CCDC references: 2564329, 2564328, 2564327, 2564326, 2564325, 2564324, 2564323, 2564322, 2564321

## Figures and Tables

**Table:** Experiments were carried out at 100 K.

	**1a**	**1c**	**1e**
Crystal data			
Chemical formula	C_7_H_8_N_4_O_2_·C_15_H_10_O_6_	2C_7_H_8_N_4_O_2_·2C_15_H_10_O_8_·H_2_O	2C_7_H_8_N_4_O_2_·C_16_H_14_O_6_
*M*_r_	466.41	1014.82	662.62
Crystal system, space group	Monoclinic, *P*2_1_/*n*	Monoclinic, *P*2_1_/*c*	Triclinic, *P* 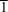
*a*, *b*, *c* (Å)	6.51941 (9), 7.77386 (10), 39.0816 (5)	7.3270 (1), 32.4057 (4), 17.8062 (2)	6.8286 (2), 14.9790 (3), 16.0763 (4)
α, β, γ (°)	90, 92.4920 (12), 90	90, 97.827 (1), 90	88.624 (2), 83.176 (2), 87.020 (2)
*V* (Å^3^)	1978.82 (5)	4188.46 (9)	1630.25 (7)
*Z*	4	4	2
Radiation type	Cu *K*α	Cu *K*α	Cu *K*α
μ (mm^−1^)	1.03	1.12	0.88
Crystal size (mm)	0.2 × 0.2 × 0.2	0.1 × 0.02 × 0.01	0.05 × 0.03 × 0.01

Data collection			
Diffractometer	Agilent SuperNova Dual Source diffractometer with an Atlas detector	Agilent SuperNova Dual Source diffractometer with an Atlas detector	Agilent SuperNova Dual Source diffractometer with an Atlas detector
Absorption correction	Multi-scan (*CrysAlis PRO*; Rigaku OD, 2023 ▸)	Multi-scan (*CrysAlis PRO*; Rigaku OD, 2022 ▸)	Multi-scan (*CrysAlis PRO*; Rigaku OD, 2023 ▸)
*T*_min_, *T*_max_	0.807, 1.000	0.568, 1.000	0.935, 1.000
No. of measured, independent and observed reflections	13045, 4130, 3800 [*I* ≥ 2σ(*I*)]	25287, 8359, 5887 [*I* > 2σ(*I*)]	26501, 6746, 6048 [*I* > 2σ(*I*)]
*R*_int_	0.023	0.055	0.029
(sin θ/λ)_max_ (Å^−1^)	0.633	0.625	0.632

Refinement			
*R*[*F*^2^ > 2σ(*F*^2^)], *wR*(*F*^2^), *S*	0.038, 0.095, 1.06	0.049, 0.118, 0.99	0.048, 0.125, 1.05
No. of reflections	4130	8359	6746
No. of parameters	329	726	469
No. of restraints	0	0	3
H-atom treatment	H atoms treated by a mixture of independent and constrained refinement	H atoms treated by a mixture of independent and constrained refinement	H atoms treated by a mixture of independent and constrained refinement
Δρ_max_, Δρ_min_ (e Å^−3^)	0.33, −0.27	0.58, −0.27	0.26, −0.19

**Table:** 

	**2a**	**2c**	**3a**
Crystal data			
Chemical formula	C_7_H_7_ClN_4_O_2_·C_15_H_10_O_6_	C_7_H_7_ClN_4_O_2_·C_15_H_10_O_8_	C_7_H_7_BrN_4_O_2_·C_15_H_10_O_6_
*M*_r_	500.85	532.84	545.30
Crystal system, space group	Monoclinic, *P*2_1_/*n*	Triclinic, *P* 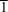	Monoclinic, *P*2_1_/*n*
*a*, *b*, *c* (Å)	10.14353 (15), 19.7196 (3), 10.42202 (19)	7.7588 (5), 10.6363 (7), 13.4366 (7)	10.1578 (1), 19.8059 (3), 10.4534 (1)
α, β, γ (°)	90, 93.0362 (14), 90	78.246 (5), 81.294 (5), 81.946 (5)	90, 92.539 (1), 90
*V* (Å^3^)	2081.75 (6)	1066.24 (12)	2101.00 (4)
*Z*	4	2	4
Radiation type	Cu *K*α	Cu *K*α	Cu *K*α
μ (mm^−1^)	2.18	2.24	3.22
Crystal size (mm)	0.2 × 0.08 × 0.04	0.22 × 0.15 × 0.1	0.2 × 0.1 × 0.08

Data collection			
Diffractometer	Agilent SuperNova Dual Source diffractometer with an Atlas detector	Agilent SuperNova Dual Source diffractometer with an Atlas detector	Agilent SuperNova Dual Source diffractometer with an Atlas detector
Absorption correction	Multi-scan (*CrysAlis PRO*; Rigaku OD, 2022 ▸)	Multi-scan (*CrysAlis PRO*; Rigaku OD, 2022 ▸)	Multi-scan (*CrysAlis PRO*; Rigaku OD, 2022 ▸)
*T*_min_, *T*_max_	0.705, 1.000	0.481, 1.000	0.871, 1.000
No. of measured, independent and observed reflections	12088, 4177, 3533 [*I* > 2σ(*I*)]	6593, 4156, 3656 [*I* > 2σ(*I*)]	12439, 4220, 3606 [*I* > 2σ(*I*)]
*R*_int_	0.030	0.029	0.029
(sin θ/λ)_max_ (Å^−1^)	0.626	0.626	0.625

Refinement			
*R*[*F*^2^ > 2σ(*F*^2^)], *wR*(*F*^2^), *S*	0.036, 0.100, 1.03	0.038, 0.105, 1.02	0.032, 0.086, 1.06
No. of reflections	4177	4156	4220
No. of parameters	338	364	334
No. of restraints	0	0	4
H-atom treatment	H atoms treated by a mixture of independent and constrained refinement	H atoms treated by a mixture of independent and constrained refinement	H atoms treated by a mixture of independent and constrained refinement
Δρ_max_, Δρ_min_ (e Å^−3^)	0.25, −0.38	0.33, −0.38	0.42, −0.43

**Table:** 

	**3c**	**3a′**	**a·H_2_O**
Crystal data			
Chemical formula	C_7_H_7_BrN_4_O_2_·C_15_H_10_O_8_	C_7_H_7_BrN_4_O_2_·C_15_H_10_O_6_·CH_4_O	C_15_H_10_O_6_·H_2_O
*M*_r_	577.30	577.35	304.25
Crystal system, space group	Triclinic, *P* 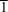	Triclinic, *P* 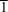	Monoclinic, *C*2/*c*
*a*, *b*, *c* (Å)	7.7841 (3), 10.7050 (5), 13.4516 (6)	9.9847 (5), 10.6290 (4), 12.1537 (4)	27.7113 (11), 3.7151 (2), 24.7282 (11)
α, β, γ (°)	78.741 (4), 81.454 (4), 81.657 (4)	67.509 (4), 81.274 (3), 73.675 (4)	90, 98.208 (2), 90
*V* (Å^3^)	1079.31 (8)	1142.27 (9)	2519.7 (2)
*Z*	2	2	8
Radiation type	Cu *K*α	Mo *K*α	Ga *K*α, λ = 1.34139 Å
μ (mm^−1^)	3.25	1.86	0.70
Crystal size (mm)	0.09 × 0.06 × 0.05	0.3 × 0.2 × 0.1	0.03 × 0.03 × 0.03

Data collection			
Diffractometer	Agilent SuperNova Dual Source diffractometer with an Atlas detector	Agilent SuperNova Dual Source diffractometer with an Atlas detector	Bruker APEXII CCD
Absorption correction	Multi-scan (*CrysAlis PRO*; Rigaku OD, 2023 ▸)	Multi-scan (*CrysAlis PRO*; Rigaku OD, 2023 ▸)	Multi-scan (*SADABS*; Bruker, 2016 ▸)
*T*_min_, *T*_max_	0.899, 1.000	0.615, 1.000	0.658, 0.751
No. of measured, independent and observed reflections	6335, 4197, 3614 [*I* > 2σ(*I*)]	7430, 4532, 4048 [*I* > 2σ(*I*)]	11000, 2557, 1890 [*I* > 2σ(*I*)]
*R*_int_	0.026	0.035	0.041
(sin θ/λ)_max_ (Å^−1^)	0.626	0.622	0.626

Refinement			
*R*[*F*^2^ > 2σ(*F*^2^)], *wR*(*F*^2^), *S*	0.036, 0.097, 1.04	0.029, 0.066, 1.05	0.040, 0.110, 1.06
No. of reflections	4197	4532	2557
No. of parameters	342	358	224
No. of restraints	0	0	0
H-atom treatment	H-atom parameters constrained	H atoms treated by a mixture of independent and constrained refinement	H atoms treated by a mixture of independent and constrained refinement
Δρ_max_, Δρ_min_ (e Å^−3^)	1.08, −0.58	0.49, −0.41	0.19, −0.17

Computer programs: *CrysAlis PRO* (Rigaku OD, 2022 ▸, 2023 ▸), *APEX2* (Bruker, 2016 ▸), *SAINT* (Bruker, 2016 ▸), *olex2.solve* (Bourhis *et al.*, 2015 ▸), *SHELXT2018/2019* (Sheldrick, 2015*a* ▸), *olex2.refine* (Bourhis *et al.*, 2015 ▸), *SHELXL2019* (Sheldrick, 2015*b* ▸) and *OLEX2* (Dolomanov *et al.*, 2009 ▸).

**Table 2:** Theophylline (Tph)–flavonoid co­crys­tal phases

Phase	Formula	Cocrystal System	Comments	Reference
**1a**	C_22_H_18_N_4_O_8_	Tph–kaempferol	*R*_1_ = 3.78%	This work
**1b**	C_22_H_18_N_4_O_9_·0.5H_2_O	Tph–quercetin	0.5H_2_O	*a*
**1c**	C_22_H_18_N_4_O_10_·0.5H_2_O	Tph–myricetin	0.5H_2_O, *R*_1_ = 4.87%	This work
**1d**	C_29_H_26_N_8_O_9_·3H_2_O	Tph–baicalein (2/1)	3H_2_O	*b*
**1e**	C_30_H_30_N_8_O_10_·*x*H_2_O	Tph–*rac*-hesperetin (2/1)	Solvate, *R*_1_ = 4.85	This work
**1f**	C_22_H_20_N_4_O_10_·CH_3_CN	Tph–di­hydro­myricetin	MeCN solvate	*c*

References: (*a*) Wang *et al.* (2022 ▸) (CSD refcode JATPIH); (*b*) Zhu *et al.* (2017 ▸) (CSD refcode KAMQIB); (*c*) Sun *et al.* (2023 ▸) (CSD refcode NEXSIW).

**Table 3:** 8-Halotheo­phyl­line (8-*X*-Tph)–flavonoid co­crys­tal phases reported in this article

Phase	Formula	Cocrystal system	Comments
**2a**	C_22_H_17_ClN_4_O_8_	8-Cl-Tph–kaempferol	*R*_1_ 3.61%
**2c**	C_22_H_17_ClN_4_O_10_	8-Cl-Tph–myricetin	*R*_1_ 3.82%
**3a**	C_22_H_17_BrN_4_O_8_	8-Br-Tph–kaempferol	*R*_1_ 3.18%
**3a′**	C_22_H_17_BrN_4_O_8_·CH_4_O	8-Br-Tph–kaempferol MeOH	*R*_1_ 2.86%
**3c**	C_22_H_17_BrN_4_O_10_	8-Br-Tph–myricetin	*R*_1_ 3.86%

**Table 4:** Specific volumes for theophylline–flavonoid coformer/cocrystal phases

(*a*) specific volumes for theophylline–flavonoid coformer phases				
Phase	Formula	Unit cell volume (Å^3^), *Z*	Mol­ecular volume (anhydrous)^ii^ (Å^3^)	Reference/CSD refcode
**1**, theophylline (Tph)	C_7_H_8_N_4_O_2_	390.9, 2	195.5 (195.5)	Ye *et al.* (2025 ▸)
**2**, 8-Cl-theophylline (8-Cl-Tph)	C_7_H_7_ClN_4_O_2_	418.0, 2	209.0 (209.0)	Ye *et al.* (2025 ▸)
**3**, 8-Br-theophylline (8-Br-Tph)	C_7_H_7_BrN_4_O_2_	860.9, 4	215.2 (215.2)	Ye *et al.* (2025 ▸)
**a**, kaempferol, H_2_O (Kmp)	C_15_H_10_O_6._H_2_O	2519.7, 8	315.0 (295.0)	This work
**b**, quercetin, H_2_O (Que)	C_15_H_10_O_7._H_2_O	1273.8, 4	318.4 (298.4)	AKIJEK
**c**, myricetin, H_2_O (Myr)^i^	C_15_H_10_O_8._H_2_O	1305.5, 4	323.2 (303.2)	NIKLAX
**d**, baicalein (Bai)	C_15_H_10_O_5_	1170.7, 4	292.7 (292.7)	RAMGOB01
**e**, hesperetin (Hes)	C_16_H_14_O_6_	1359.5, 4	339.9 (339.9)	YEHROS
**f**, di­hydro­myricetin, 2H_2_O (Dhm)	C_15_H_10_O_8._2H_2_O	2928.6, 8	366.1 (326.1)	SIMVOA02
				
(*b*) specific volumes for theophylline–flavonoid cocrystal phases				
Phase	Unit cell volume (Å^3^), *Z*	Formula volume (anhydrous)*^*b*^* (Å^3^)	Coformer volume (anhydrous) (ΔV Å^3^)	Reference/CSD refcode
**1a**, Tph–Kmp (1/1)	1978.8, 4	494.7 (494.7)	490.5, −4.2	This work
**1b**, Tph–Que (1/1), 0.5H_2_O	4083.2, 8	510.4 (500.4)	493.9, −5.1	JATPIH
**1c**, Tph–Myr (1/1), 0.5H_2_O	4188.5, 8	523.6 (513.6)	498.7*^*a*^*, −14.9	This work
**1d**, Tph–Bai (2/1), 3H_2_O	1496.2, 2	748.1 (688.1)	683.6, −4.7	KAMQIB
**1e**, Tph–Hes (2/1) MeOH, 2H_2_O	1630.2, 2	815.1 (735.1)	730.8, −4.3	This work
**1f**, Tph–Dhm (1/1), MeCN	2402.0, 4	600.5 (540.5)	521.6, −18.9	NEXSIW
**2a**, 8-Cl-Tph–Kmp	2081.8, 4	520.4 (520.4)	504.0, −16.4	This work
**2c**, 8-Cl-Tph–Myr	1066.2, 2	533.1 (533.1)	512.2, −20.9	This work
**3a**, 8-Br-Tph–Kmp	2101.0, 4	525.3 (525.3)	510.2, −15.1	This work
**3a′**, 8-Br-Tph–Kmp, MeOH	1142.3, 2	571.2 (531.2)	510.2, −21.2	This work
**3c**, 8-Br-Tph–Myr	1079.3, 2	539.7 (539.7)	518.4, −21.3	This work

Notes: (i) all unit-cell data at 100 K, except for Myr·H_2_O (140 K), the coformer volume is reduced by 1% to approximate a 100 K structure; (ii) mol­ecular volumes subtracted: H_2_O = 20 Å^3^, MeOH 40 Å^3^ and MeCN 60 Å^3^. References for earlier CSD structures: AKIJEK (Domagała *et al.*, 2011 ▸), NIKLAX (Ren *et al.*, 2019 ▸), RAMGOB01 (Rossi *et al.*, 2001 ▸), YEHROS (Maeda *et al.*, 1994 ▸), SIMVOA02 (Hu *et al.*, 2020 ▸), JATPIH (Wang *et al.*, 2022 ▸), KAMQIB (Zhu *et al.*, 2017 ▸) and NEXSIW (Sun *et al.*, 2023 ▸). Except for NIKLAX, these were all redetermined by us at 100 K.

**Table 5:** Hydrogen bonds (HBs) for selected crystals and cocrystals

Phase	No. of donors, HBs	Strongest HBs (Å)	Comment	Reference
**1**, Tph	1, 1	NH⋯N 2.820 (4)	chain	Ye *et al.* (2025 ▸)
**2**, 8-Cl-Tph	1, 1	NH⋯O 2.723 (2)	dimer	Ye *et al.* (2025 ▸)
**3**, 8-Br-Tph	1, 1	NH⋯O 2.760 (2)	dimer	Ye *et al.* (2025 ▸)
**a·H_2_O**, Kmp H_2_O	6, 6	OH⋯O 2.742 (2)	4 HBs with H_2_O	This work
**1a**, Tph–Kmp	5, 5	OH⋯O 2.680 (2)	3 HBs Kmp-to-Tph	This work
		OH⋯N9 2.794 (2)	1 HB Tph-to-Kmp	
**2a**. 8-Cl-Tph–Kmp	5, 5	NH⋯O6 2.667 (2)	2 HBs Kmp-to-8-Cl-Tph	This work
		OH⋯O2 2.774 (2)	OH⋯N9 2.866 Å (weak)	
**3a**, 8-Br-Tph–Kmp	5, 5	NH⋯O6 2.671 (2)	2 HBs Kmp-to-8-Br-Tph	This work
		OH⋯O2 2.769 (2)	OH⋯N9 2.881 Å (weak)	
**3a′**, 8-Br-Tph–Kmp MeOH	6, 6	NH⋯O6 2.698 (2)	1 HB Kmp-to-8-Br-Tph	This work
		OH⋯O2 2.708 (2)	No use of N9 acceptor	
**1c**, Tph–Myr 0.5H_2_O	8, 7	OH⋯N9 2.744 (3)	H_2_O single donor	This work
**2c**, 8-Cl-Tph–Myr	7, 7	OH⋯O2 2.689 (2)	2 HBs Kmp-to-8X-Tph	This work
			No use of N9 acceptor	
**3c**, 8-Br-Tph–Myr	7, 7	OH⋯O2 2.652 (2)	2 HBs Kmp-to-8X-Tph	This work
			No use of N9 acceptor	

**Table 6:** Microwave-assisted formation for selected cocrystals*

Phase	Alkaloid *M*_r_ (mg)	Cocrystal *M*_r_	% yield
	Flavonoid *M*_r_ (mg)	Yield (mg)	
**1a**, Tph–Kmp (1/1)	180.2, 180	466.4	88.6
	286.2, 300	413	
**1b**, Tph–Que (1/1), 0.5H_2_O	180.2, 180	491.4	91.8
	302.2, 310	451	
**1c**, Tph–Myr (1/1), 0.5H_2_O	180.2, 180	507.4	86.3
	318.2, 325	438	
**1d**, Tph–Bai (2/1), 3H_2_O	180.2, 270 (1.5 mmol)	648.6	85.9
	270.2, 210 (0.75 mmol)	557	
**2a**, 8-Cl-Tph–Kmp (1/1)	214.6, 215	500.8	86.5
	286.2, 300	433	
**2c**, 8-Cl-Tph–Myr (1/1)	214.6, 215	532.8	92.0
	318.2, 325	490	
**3a**, 8-Br-Tph–Kmp (1/1)	259.1, 130	545.3	80.7
	286.2, 150	220	
**3c**, 8-Br-Tph–Myr (1/1)	259.1, 130	577.3	88.5
	318.2, 160	255.5	

Note: (*) microwave-assisted cocrystal formation in Biotage Initiator+ using sealed 2–5 ml reaction vials; 1 mmol scale in 2 ml 1-butanol, except for **1d**, a 2:1 cocrystal on a 0.75 mmol scale in 2 ml 1-butanol, and **3a** and **3c** on a 0.5 mmol scale in 1 ml 1-butanol.
